# Physician job satisfaction as a public health issue

**DOI:** 10.1186/2045-4015-1-51

**Published:** 2012-12-14

**Authors:** Richard L Kravitz

**Affiliations:** 1 UC Davis Division of General Medicine, 4150 V. Street, Suite 2400 PSSB, Sacramento, CA, 95817, USA

## Abstract

In Hirschman’s classic formulation, physicians can signal discontent with their conditions of work by “exiting” (leaving the profession or not entering it in the first place) or by giving “voice” to their concerns (e.g. complaining, protesting, bargaining collectively, or conducting work actions and strikes). This Commentary reviews the findings of a survey of Israeli neonatologists by Moshe et al. Survey respondents were satisfied with their careers but not with salary, patient care demands, and leisure time, a pattern that has been seen in other countries, particularly within “small, acute care specialties” (SACS). One question for policymakers is how to help physicians in SACS maintain work-life balance and avoid burnout while providing superb patient care. The Commentary considers several possible solutions while advocating for rigorous and comprehensive monitoring of physician satisfaction over time.

## 

Physician job satisfaction is a fraught topic. After all, doctors are among the more highly paid and most respected members of society in most developed countries. Their role is to promote patients’ welfare, not their own. Medical professionalism and training honors stoic acceptance of duty and eschews whining. On the other hand, no sick person wants to be seen by a grumpy doctor. And deep down, the public knows it would be unwise to rely on a chronically demoralized physician workforce, just as it would be unwise to fly in airplanes flown by disgruntled pilots. Therefore, although to say so triggers some embarrassment in medical circles, physician satisfaction is a public health issue.

When physicians are dissatisfied with their conditions of practice, they have two broad options. Adopting Hirschman’s classic formulation [1], physicians may either exit (abandon their current job or the profession) or give voice to their concerns (by complaining individually or organizing collectively). For physicians, “exit” strategies could manifest as abandoning plans to attend medical school or to train in a particular specialty, switching specialties, reducing clinical work in favor of research or administration, or retiring early. These passive responses address burnout at the individual level but do nothing to call attention to the underlying determinants of distress. On the other hand, “voice” strategies such as collective bargaining and strikes can be disruptive (and in some cases, unethical) [2], but they do tend to flush conflict and distress into the open, which can sometimes be salutory.

The paper by Moshe et al. in this issue of IJHPR [3] focuses on the work satisfaction of neonatologists. This specialty is responsible for the care of some of our tiniest, most vulnerable patients. Little of what neonatologists do can be undertaken by general pediatricians or by other pediatric subspecialists. At the same time, the neonatology workforce in many countries (including Israel) is limited. When growing clinical demands collide with staffing shortages, physician satisfaction is a victim.

Given the steep increase in demand for neonatology services in Israel over the past decade (fueled by a high and growing birthrate), the results of the paper by Moshe et al. are generally reassuring. This survey of 112 neonatologists (representing a remarkably high proportion of both staff physicians and post-residency fellows throughout the country) found that respondents derived considerable satisfaction from their careers and from patient care. These results are consistent with Leigh et al., who found in a US national survey that neonatologists were among the physicians most satisfied with their overall careers [4]. However, whereas Leigh et al. asked a single question, Moshe et al. dug deeper, finding that Israeli neonatologists were somewhat *dissatisfied* with salary, workload, and leisure time. This general pattern of results is similar to that found in other studies of physician satisfaction across a broad range of specialties [5,6].

However, like other practitioners in “small, acute care subspecialties” (SACS) such as neurosurgery, cardiothoracic surgery, and perinatology, neonatologists are particularly vulnerable to workload pressures. This is because they are few in number and have specialized, non-substitutable skills that may be needed emergently. A group of 3 perinatologists may function fine under ordinary conditions but not so well when one is sick or on vacation – or during times of unusually heavy patient demand. This lack of surge capacity may be experienced as stress and contribute to burnout. If burnout leads to “exit,” the supply/demand mismatch is only exacerbated.

One question for policymakers is how to help physicians in SACS maintain work-life balance and avoid burnout while providing superb patient care. One approach is to increase physician supply in vulnerable specialties through increased fellowship funding. However, this particular instrument is blunt, slow, and expensive. Another is to increase use of midlevel providers for routine aspects of care and even some technical procedures. Some U.S. hospitals have successfully deployed neonatal nurse practitioners to share call responsibilities, sometime supported by telemedicine. Finally, large urban regions or even small countries such as Israel could establish programs in which neonatologists are shared across hospitals, which would allow more flexibility in scheduling and greater surge capacity.

Physician satisfaction is neither unidimensional nor static; it can change as conditions of practice evolve. In Israel, the new physician collective bargaining agreement signed toward the end of 2011 includes new financial incentives for physicians practicing in the periphery of the country as well as increased pay for physicians in “distressed specialties,” including neonatology. Following implementation of these incentives, additional neonatology training slots have been established. As a result of these and other developments, it would be reasonable for Moshe et al. to repeat their survey in several years. In fact, a strong argument could be made for systematically measuring physician satisfaction comprehensively on a periodic basis, not just in Israel but throughout the world. Periodic surveys of physician satisfaction in the manner of Moshe et al. would give voice to distressed specialties, possibly allowing for redress of grievances without need for more extreme measures. Of course, health care system leaders must balance doctor satisfaction against the public interest. But they ignore the well-being of health professionals at their peril.

A strong physician workforce that is deeply engaged with the work of healing and includes primary care physicians, specialists, and practitioners of small, acute care subspecialities is essential for delivering quality care. Moshe et al. remind us that we must continue to seek innovative solutions that fit the local landscape. The alternative is to risk physician discontent, burnout, and diminished engagement with practice. And that means bumpy skies ahead.

## Competing interests

The author declares that he has no competing interests.

## Authors’ information

The author is Professor of Internal Medicine (General Medicine) and Co-Vice Chair for Research at the University of California, Davis. He also serves as Co-Editor-in-Chief of the Journal of General Internal Medicine, the official journal of the Society of General Internal Medicine.

## Commentary prepared in response to

Moshe M, Perry ZH, Salzer L, Zemora E, Toker A. Work satisfaction, quality of life, and leisure time of residents and senior neonatologists in Israel.

## References

[B1] Hirschman AO Exit, Voice, and Loyalty: responses to decline in firms, organizations and states 1970 Cambridge: Harvard University Press

[B2] Kravitz RL Linn L Tennant N Adkins E Zawacki B To strike or not to strike? House-staff attitudes and behaviors during a hospital work action West J Med 1990 153 515 519 2260287 PMC1002602

[B3] Moshe M Perry ZH Salzer L Toker A Zmora E Work satisfaction, quality of life, and leisure time of neonatology fellows and senior neonatologists in Israel Isr J of Health Policy Res 2012 1 50 23241385 10.1186/2045-4015-1-50 PMC3565952

[B4] Leigh JP Tancredi DJ Kravitz RL Physician career satisfaction within specialties BMC Health Serv Res 2009 9 166 10.1186/1472-6963-9-166 19758454 PMC2754441

[B5] Kravitz RL Linn LS Shapiro MF Physician satisfaction under the Ontario Health Insurance Plan Med Care 1990 28 502 512 10.1097/00005650-199006000-00003 2355756

[B6] Bovier PA Perneger TV Predictors of work satisfaction among physicians Eur J Public Health 2003 13 299 305 10.1093/eurpub/13.4.299 14703315

